# MiR-320 regulates cardiomyocyte apoptosis induced by ischemia–reperfusion injury by targeting AKIP1

**DOI:** 10.1186/s11658-018-0105-1

**Published:** 2018-08-28

**Authors:** Zhi-Qiang Tian, Hong Jiang, Zhi-Bing Lu

**Affiliations:** 1 Department of Cardiology, Inner Mongolia People’s Hospital, Hohhot, 010017 People’s Republic of China; 2 Department of Cardiology, Remin Hospital of Wuhan University, 238 Jiefang Road, Wuhan, 430060 People’s Republic of China

**Keywords:** microRNA-320, Ischemia–reperfusion, AKIP1, Cardiomyocyte apoptosis

## Abstract

**Background:**

MicroRNAs play important roles in regulation of the cardiovascular system. The purpose of this study was to investigate microRNA-320 (miR-320) expression in myocardial ischemia-reperfusion (I/R) injury and the roles of miR-320 in cardiomyocyte apoptosis by targeting AKIP1 (A kinase interacting protein 1).

**Methods:**

The level of miR-320 was detected using quantitative real-time polymerase chain reaction (qRT-PCR), and cardiomyocyte apoptosis was detected via terminal dUTP nick end-labeling assay. Cardiomyocyte apoptosis and the mitochondrial membrane potential were evaluated via flow cytometry. Bioinformatics tools were used to identify the target gene of miR-320. The expression levels of AKIP1 mRNA and protein were detected via qRT-PCR and Western blot, respectively.

**Results:**

Both the level of miR-320 and the rate of cardiomyocyte apoptosis were substantially higher in the I/R group and H9c2 cells subjected to H/R than in the corresponding controls. Overexpression of miR-320 significantly promoted cardiomyocyte apoptosis and increased the loss of the mitochondrial membrane potential, whereas downregulation of miR-320 had an opposite effect. Luciferase reporter assay showed that miR-320 directly targets AKIP1. Moreover, knock down and overexpression of AKIP1 had similar effects on the H9c2 cells subjected to H/R.

**Conclusions:**

miR-320 plays an important role in regulating cardiomyocyte apoptosis induced by I/R injury by targeting AKIP1 and inducing the mitochondrial apoptotic pathway.

**Electronic supplementary material:**

The online version of this article (10.1186/s11658-018-0105-1) contains supplementary material, which is available to authorized users.

## Background

Despite current optimal treatment, ischemic heart disease (IHD) remains a leading cause of mortality [1]. The main therapeutic method for IHD is timely reperfusion of the occluded artery; however, reperfusion may lead to myocardial ischemia–reperfusion (I/R) injury [2]. Accumulating evidence indicates that myocardial I/R injury may induce cardiomyocyte apoptosis, which suggests that apoptosis plays an important role in the development of myocardial diseases [3]. In addition, a previous study indicated that improvements in metabolic recovery in cardiomyocytes and mitochondrial-derived oxidative stress are crucial for prevention of myocardial I/R injury [4]. Thus, the elucidation of the endogenous defensive molecular mechanisms in myocardial I/R injury will definitely provide a novel therapeutic strategy for ischemic heart disease.

MicroRNAs (miRNAs) are small non-coding molecules that negatively regulate gene expression at the post-transcriptional level by binding to target messenger RNAs [5, 6]. Recent studies have shown that miRNAs control approximately 60% of protein-coding genes, which suggests that miRNAs are involved in a substantial number of biological processes, including cell growth, apoptosis and differentiation [7, 8]. Studies have demonstrated that miRNAs play crucial roles in the development of myocardial diseases, including myocardial I/R injury [9–11]. The present study aimed to investigate the potential roles and mechanisms of miR-320 in cardiomyocyte apoptosis induced by myocardial I/R injury, thus providing novel therapeutic strategies for the treatment of myocardial I/R injury. We determined that miR-320 was significantly upregulated in the myocardial tissues of mice after I/R injury and H9c2 cells subjected to H/R. Ectopic expression of miR-320 promoted cardiomyocyte apoptosis and enhanced the mitochondrial membrane potential, whereas the miR-320 inhibitor resulted in the opposite phenomenon in H9c2 cells subjected to H/R. Furthermore, we determined that AKIP1 is one direct target gene of miR-320 and confirmed that miR-320 may exert its effects on the promotion of cardiomyocyte apoptosis by downregulating AKIP1 in H9c2 cells subjected to H/R. Therefore, these results support an in-depth understanding of the roles and mechanisms of miR-320 in the regulation of I/R injury-induced cardiomyocyte apoptosis and cardiac function, thus emerging as underlying therapeutic agents for myocardial I/R injury.

## Methods

### Animals and treatments

Thirty healthy male C57BL/6 mice (8–10 weeks old) that weighed 20–30 g were maintained in an environmental room with a constant temperature of 20°C, humidity of 60% and a programmed 12 h light / 12 h dark cycle for circadian control. All mice were purchased from Beijing Laboratorial Animal Center. The animal experiment was approved by the Laboratory Animal Ethics Committee of Wuhan University (Wuhan, People’s Republic of China [PRC]). Reasonable efforts were made to minimize animal suffering. All mice were allowed free access to drinking water and a sterilized standard diet. The mice were randomly separated into two groups: the sham group (*n* = 15): the mice underwent a sham operation with hearts enlarged and a suture under the left anterior descending coronary artery (LAD), without ligation of the coronary artery for 150 min; the I/R group (*n* = 15): the mice were treated with the LAD, which was reversibly ligated, and I/R was induced by 30 min ischemia followed by 2 h reperfusion.

### I/R injury model

All mice were anesthetized with sodium pentobarbital (50 mg/kg) via intraperitoneal injection and were fixed for endotracheal intubation using a small animal respirator. The skin was disinfected with iodine after removing hair from the precordium. A longitudinal incision was made from the third to fourth ribs, separating the pectoralis major muscle, serratus anterior muscle and pectoralis minor muscle, and exposing the heart. A 6–0 nylon suture was subsequently placed around l-2 cm of the root of the LAD. The suture was loosened after occlusion for 30 min, which was followed by 2 h reperfusion of the LAD. The hearts of all mice were subsequently separated and prepared for the following experiments. The electrocardiogram (ECG) was synchronously recorded until the end of the experiment.

### Infarct size determination

Infarct size of the myocardium was measured by triphenyltetrazolium chloride (TTC, Sigma-Aldrich, USA) staining. The hearts were perfused with saline on a Langendorff system to wash blood from the coronary vasculature. Two percent Evans blue dye (wt/vol, Sigma-Aldrich, USA) was injected into the vena cava to identify the area of myocardial perfusion at the end of the myocardial reperfusion. The left ventricle was isolated by removing the right ventricle and washing out the remaining blood; the sample was then cut into slices and stained with 1% TTC for 10 min at 37°C. The red region was identified as the area at risk (AAR), whereas the infarct area (IA) was identified by a non-staining (white) region. The AAR and IA were determined by computerized planimetry. Finally, the percentage of IA/AAR was calculated..

### TUNEL assay

Terminal dUTP nick end-labeling (TUNEL) staining was performed with an in situ cell death detection kit (Promega, USA) in accordance with the manufacturer’s protocol to detect apoptotic cardiomyocytes. The extracted tissue from the mice with the I/R model was fixed using 10% formic acid solution. The fixation lasted for 24 h at room temperature. Paraffin-embedded specimens were subsequently sliced, deparaffinized and dehydrated. After washing with phosphate buffer solution (PBS) twice, the sections were blocked in 0.1% Triton X-100 and 0.1% sodium citrate for 15 min. The sections were subsequently washed with PBS three times and incubated in 50 μL of TUNEL reaction mixture (Roche, Switzerland), which contained 0.3 U/mL of terminal deoxynucleotidyl transferase and 6.66 mM/mL of biotin dUTP in a moist chamber at 37°C for 1.5 h; the sections were washed with PBS, incubated with converter-peroxidase at 37°C for 30 min, and incubated with diaminobenzidine for 10 min. The sections were then washed with PBS three times and stained with hematoxylin prior to observation under a light microscope.

### Cell culture and transfection

Rat cardiomyocyte H9c2 cells were grown in RPMI 1640 (GIBCO BRL, USA) supplemented with 10% fetal bovine serum, 100 IU/ml of penicillin and 100 μg/ml of streptomycin, followed by incubation at 37°C in a humidified chamber supplemented with 5% CO_2_. Transfection was performed with Lipofectamine 2000 Reagent (Invitrogen) following the manufacturer’s protocol. Spiked RFP-expressing vector was used to monitor the transfection efficiency.

### Hypoxia/reoxygenation injury model

Using the model of hypoxia/reoxygenation (H/R) injury, we examined the effect of hypoxia-induced and reoxygenation-induced cell death. A modular incubator (Model 3131, Forma Scientific, Marietta, USA) was used to induce hypoxia by exposing the cells to 1% O_2_, 94% N_2_, and 5% CO_2_ for 24 h. After hypoxia, the cells were exposed to 95% air and 5% CO_2_ at 37°C for 12 h. The cells in the control group were under normoxia.

### RNA isolation and quantitative real-time PCR

Total RNA from cultured cells or fresh surgical tissues was extracted using Trizol reagent (Invitrogen, USA), and qRT-PCR was performed using the All-in-One miRNA qRT-PCR detection kit (GeneCopoeia, USA) for miR-320 and small nuclear RNA U6, which was used as an endogenous control. The relative expression levels of mRNA were detected via SYBR green qRT-PCR assays (Bio-Rad Laboratories Inc., USA), and β-actin was used as an endogenous control. All qRT-PCR was performed on an ABI 7500 thermocycler (Thermo Fisher Scientific, USA). The primer sequences are shown in Table 1. The relative expression levels were calculated using the 2 ^-ΔΔct^ method.

**Table 1:** Primer sequences of miR-320, U6, AKIP1 mRNA and β-actin

Gene	Primer sequences
miR-320	Forward: 5’-TGCGGAAAAGCTGGGTTGAGAG-3′
	Reverse: 5’-CCAGTGCAGGGTCCGAGGT-3′
U6	Forward: 5’-CTCGCTTCGGCAGCACA-3′
	Reverse: 5’-AACGCTTCACGAATTTGCGT-3′
AKIP1	Forward: 5’-ATGCCAGAGGAAGGAGGAGC-3′
	Reverse: 5’-GAGCCCACAGTGACAGAATAGG-3′
β-actin	Forward: 5’-AGTGTGACGTGGACATCCGCAAAG-3′
	Reverse: 5’-ATCCACATCTGCTGGAAGGTGGAC-3’

### Construction of expression vectors

MiR-320 mimics (miR-320), mimic control (miR control), anti-miR-320 and anti-miR control were purchased from RiboBio. AKIP1-specific small interfering RNAs (siRNA-AKIP1) and the control small interfering RNAs (siRNA-control) were purchased from GeneChem (Shanghai, PRC). The coding sequence of AKIP1 was amplified and cloned into a pcDNA3.0 vector to generate AKIP1-expression vectors, and the empty pcDNA3.0 vector was used as the control. The primers used for the AKIP1 coding sequence were 5′-CGTAAGCTTGAATACTGTTCTGGCGG-3′ (forward) and 5′-ATGCCTCGAGTCACA CGGGGAAGACC-3′ (reverse).

### Flow cytometry analysis

To detect cell apoptosis, the cardiomyocytes were digested with trypsin, washed, dual stained with AV and PI, and analyzed by flow cytometry on a BD FACSCalibur (Becton Dickinson Co., USA). To assess the mitochondrial membrane potential, cells were stained with rhodamine 123 (0.1–50 μg /mL) and incubated for 10 min at 37°C in a 5% CO_2_ culture medium. After two centrifugations, the cells were resuspended in the culture medium for 1 h. The samples were measured on microscope slides (excitation, 488 nm; emission, 515 nm).

### Detection of mitochondrial membrane potential

Tetramethylrhodamine ethyl ester (TMRE) was used to detect the mitochondrial membrane potential. The enhancement of the mitochondrial permeability results in a decrease in the membrane potential, thus inhibiting the accumulation of TMRE and decreasing the fluorescence value of the mitochondrion. The cells were subsequently incubated with 50 nM TMRE at room temperature for 30 min, and the fluorescence value of TMRE was observed under a fluorescence microscope.

### Luciferase-reporter assay

The cardiomyocytes were cotransfected using 0.2 μg of luciferase reporter vector with a wild-type or mutant AKIP1 3′ UTR in 48-well plates and the miR-320 mimics or mimic control. The assay was normalized with 0.05 μg of the red fluorescent protein expression vector pDsRed2-N1 (Clontech, USA). The cells were lysed with RIPA lysis buffer (0.15 M NaCl, 0.05 M Tris/HCl pH 7.2, 1% Triton X-100, and 0.1% SDS) after 48 h. The fluorescence intensities of luciferase and red fluorescent protein were detected with an F-4500 Fluorescence Spectrophotometer (Hitachi, Japan).

### Construction of miR-320 or anti-miR-320 into lentivirus expressing system

MiR-320 or anti-miR-320 was constructed into the lentivirus expression vector using a lentivirus expressing system (Invitrogen, USA). Briefly, the oligonucleotides for miR-320 were synthesized at Integrated DNA Technologies, annealed and ligated into pcDNATM6.2-GW/ EmGFP-miR. The pcDNATM6.2-GW/EmGFP-miR cassette was subsequently transferred to pDONR221TM and ultimately pLenti6/V5-DEST by two sequential Gateway BP and LR recombinations. The lentiviral control vector contains a non-sense miR sequence that enables the formation of a pre-miRNA hairpin predicted not to target any known vertebrate gene. The viral particles were produced by third-generation packaging in 293FT cells, and the lentiviral stocks were concentrated using ultracentrifugation.

### Transfection of lentivirus expressing miR-320 or anti-miR-320 into mouse hearts in vivo

Mice were intubated and anesthetized with mechanical ventilation using 5% isoflurane. Anesthesia was maintained by inhalation of 1.5–2% isoflurane in 100% oxygen. The adequacy of anesthesia was monitored by measuring the heart rate and the response to tail stimulation. The body temperature was maintained at 37 °C by surface water heating. An incision was made in the middle of the neck, and the right common carotid artery was carefully exposed. A micro-catheter was introduced into the isolated common carotid artery and positioned into the aortic root. One hundred microliters of LmiR-320 (1 × 10^8^ PFU), LmiR control, Lanti-miR-320 or Lanti-miR control were injected through the micro-catheter. The micro-catheter was gently removed, and the common carotid artery was tightened before the skin was closed. Seven days after transfection, the hearts were harvested for the isolation of microRNAs and total RNA. The expression of miR-320 and AKIP1 mRNA in the heart tissues was examined via qRT-PCR.

### Immunohistochemistry

Tissues were fixed in 4% paraformaldehyde and cut into 4 μm sections. After dewaxing and hydration, the sections were incubated in 0.1% Triton for permeabilization. The sections were subsequently blocked with 3% BSA for 1 h at room temperature. The sections were incubated with antibody against AKIP1 (Abcam, MA, USA) at 4°C overnight and then stained with horseradish peroxidase (HRP)-conjugated secondary antibody for 1 h. Counterstaining was performed with hematoxylin.

### Western blot

Cells were lysed using the protein-extraction reagent RIPA (GIBCO BRL, USA) and were supplemented with protease inhibitors. Approximately 50 μg protein extractions were subsequently subjected to 10% sodium dodecyl sulfate polyacrylamide gel electrophoresis, for transfer onto nitrocellulose membranes (Millipore, USA), and incubated with specific primary antibodies against AKIP1, Bax, Bcl-2, cleaved caspase-3, cleaved caspase-9, p-ASK1, p-JNK, p-p38 and GAPDH (Proteintech Group, Inc., PRC). Horseradish peroxidase-conjugated goat anti-rabbit IgG (Proteintech Group, Inc., PRC) and ECL detection systems (PerkinElmer, NEL100001EA, USA) were used to visualize the specific binding.

### Statistical analysis

Data are presented as the median ± SD. Student’s t-test was performed to compare differences between two groups. Comparisons among multiple samples were performed using Statistical Analysis System software (v.9.1.3; SAS Institute, NC). *P* < 0.05 was considered statistically significant.

## Results

### MiR-320 was significantly upregulated in mouse myocardial tissues after I/R injury and H9c2 cells subjected to H/R

**Fig. 1:**
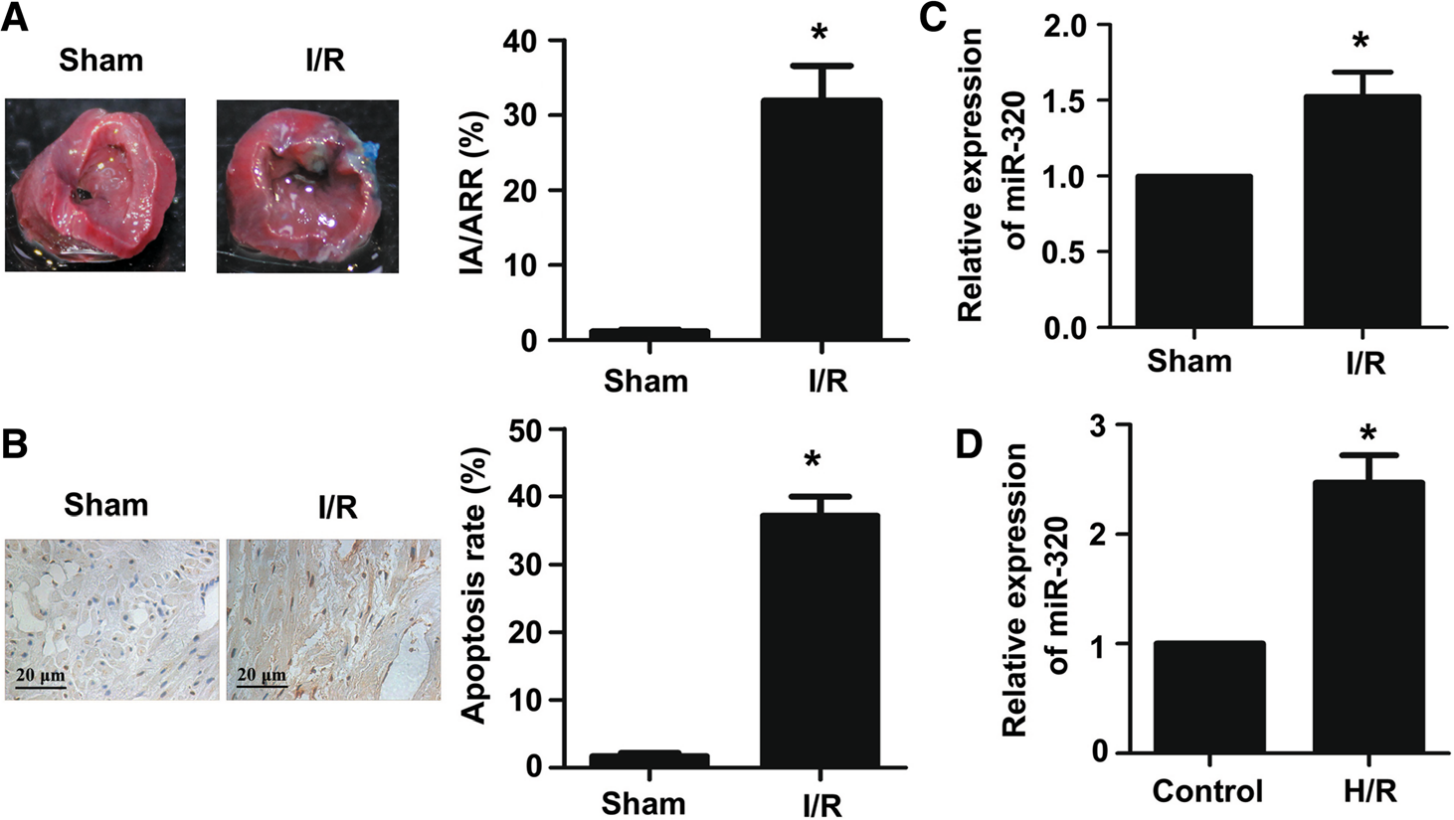
MiR-320 was significantly upregulated in mouse myocardial tissues after I/R injury and H9c2 cells subjected to H/R. **a** TTC staining was used to evaluate the myocardial infarct size. Red area, area at risk, AAR; white area, infarcted tissues. The ratio of IA (infarct area) and AAR (area at risk) was calculated and presented in the bar graph. **b** TUNEL assay was performed to evaluate the myocardial apoptosis (scale bar = 20 μm). The relative expression levels of miR-320 were evaluated via quantitative real-time PCR (qRT-PCR) in myocardial tissues under I/R treatment and the sham group (**c**); cardiomyocytes under H/R and normal condition (**d**). Each bar or point represents the mean of three independent experiments. Error bars represent SEM. *P < 0.05

To detect the extent of myocardial infarction induced by ischemia-reperfusion (I/R) injury, midventricular cross sections of TTC-stained hearts were obtained from the I/R and sham groups. As shown in Fig. 1a, the infarct size was significantly larger in the I/R group compared to the sham, which was indicated by the ratio of infarct area to area at risk (IA/AAR). Moreover, the effect of I/R injury on cardiomyocyte apoptosis was detected by TUNEL assay. As shown in Fig. 1b, the proportion of TUNEL-positive cardiomyocytes in the I/R group was clearly higher than the proportion in the sham. In previous research, we found that the relative level of miR-320 in myocardial tissues after I/R injury was significantly higher than the sham by using a miRNA microarray (Additional file 1). Furthermore, in this study, the levels of miR-320 expressed in different myocardial tissues and H9c2 cells were measured via quantitative real-time RT-PCR. The results showed that the expression levels of miR-320 were significantly higher in myocardial tissues after I/R injury and H9c2 cells subjected to H/R compared with the levels in the sham and control groups, respectively (Fig. 1c and d).

### MiR-320 promoted cardiomyocyte apoptosis induced by H/R and enhanced the loss of mitochondrial membrane potential

**Fig. 2:**
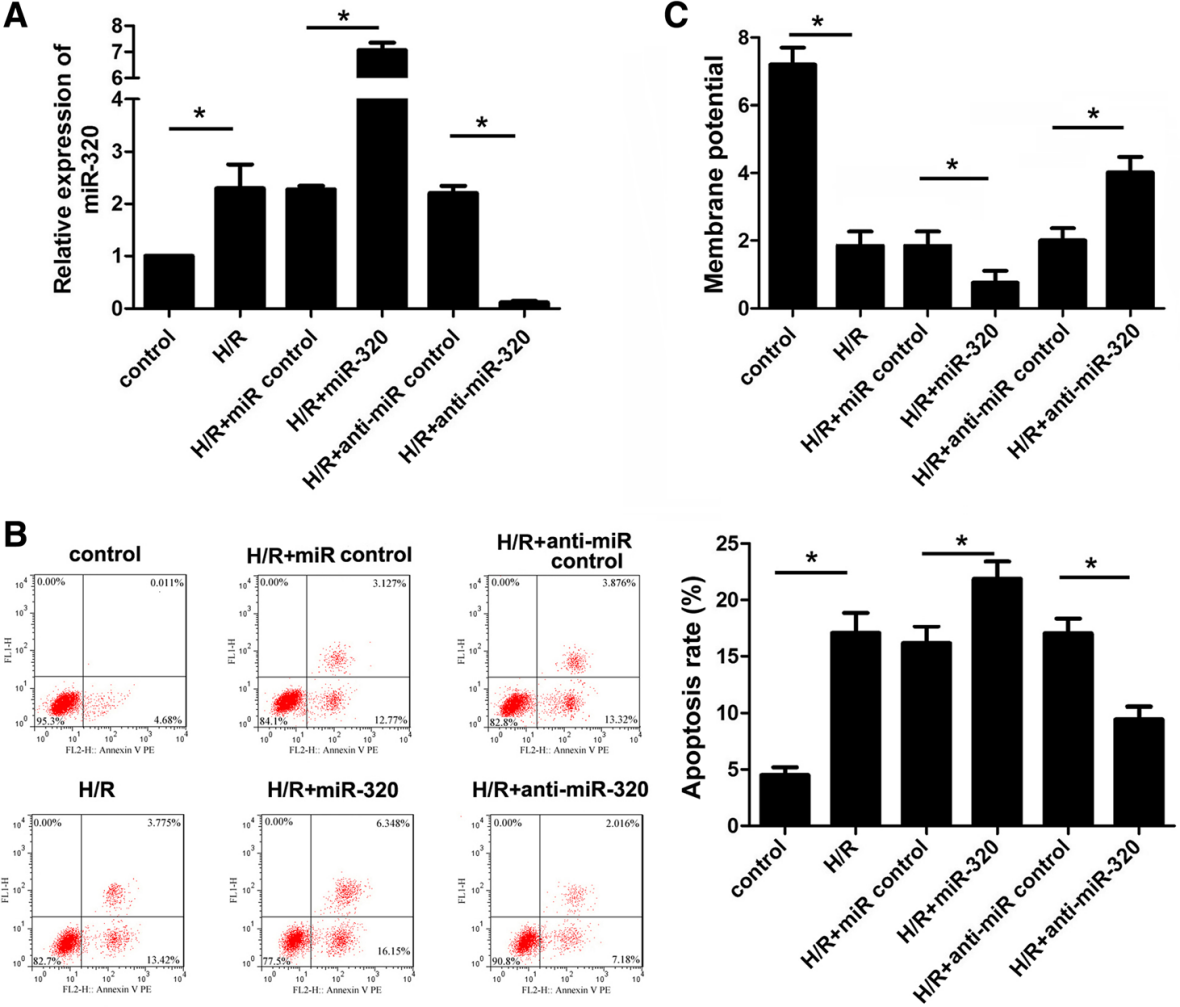
MiR-320 promoted cardiomyocyte apoptosis induced by H/R and enhanced the loss of the mitochondrial membrane potential. **a** The relative levels of miR-320 normalized to U6 in H9c2 cells subjected to H/R were detected after the cells were transfected with miR-320, anti-miR-320 or the respective control using qRT-PCR. **b** Flow cytometry was used to investigate the apoptosis of H9c2 cells. The corresponding representative micrographs are located on the left. **c** The effect of miR-320 on the mitochondrial membrane potential in cardiomyocytes was detected. *P < 0.05

To identify the effect of miR-320 on cardiomyocyte apoptosis induced by hypoxia/reoxygenation (H/R) injury, miR-320 mimics, anti-miR-320 mimics, and the corresponding negative controls were transfected to cardiomyocytes after H/R injury; qRT-PCR indicated that the miR-320 mimics significantly increased, whereas the anti-miR-320 mimics decreased the miR-320 expression in cardiomyocytes (Fig. 2a). As shown in Fig. 2b, the apoptosis rate of cardiomyocytes subjected to H/R was higher than the rate of the control cells. Moreover, the apoptosis rate of the miR-320-overexpression cardiomyocytes after H/R injury was clearly higher than the miR control group, whereas the downregulation of miR-320 inhibited the cardiomyocyte apoptosis compared to the controls as assessed via flow cytometry. In addition, mitochondrial membrane permeabilization is related to the loss of the mitochondrial membrane potential. As shown in Fig. 2c, the overexpression of miR-320 significantly enhanced the loss of the mitochondrial membrane potential; however, the loss of the mitochondrial membrane potential was attenuated by the knock down of miR-320 expression in cardiomyocytes after H/R injury. Therefore, these data suggest that miR-320 mediated the cell apoptosis of cardiomyocytes after H/R injury.

### Target gene of miR-320 analysis

**Fig. 3:**
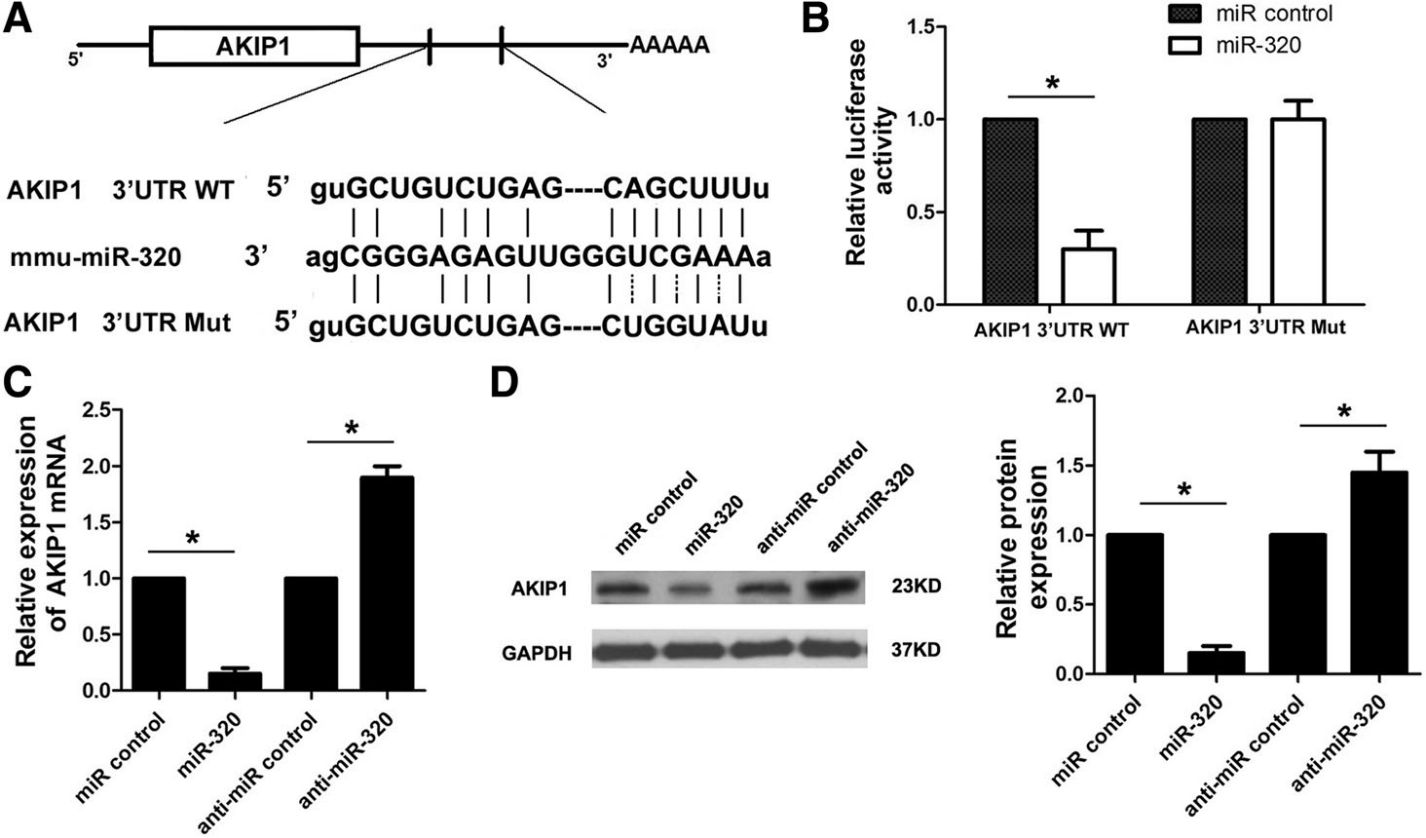
MiR-320 directly targets AKIP1. **a** Predicted binding sequences of miR-320 in AKIP1 3′ UTR. A fragment of wild-type (wt) or mutant (mut) 3′ UTR of AKIP1 was subcloned into the luciferase reporter vector. **b** H9c2 cells were co-transfected with miR-320 mimics and luciferase reporters that contained the predicted miRNA target site in the AKIP1 mRNA 3′ UTR or its corresponding mutant form. The cells co-transfected with miR control and luciferase reporters were used as the control group. The expression levels of AKIP1 mRNA and protein were measured by qRT-PCR (**c**) and Western blot (**d**), respectively. *P < 0.05

To expound the molecular mechanism that underlies miR-320 exerting its promotion effects on the cell apoptosis of cardiomyocytes after H/R injury, we predicted potential targets of miR-320 using the TargetScan and PicTar online tools and identified a conserved binding site for miR-320 in the 3′ UTR region of the AKIP1 gene. AKIP1 is a small 23 kDa protein originally identified as a breast cancer associated gene (BCA3) [12]. In humans, there are three splice variants, the full-length protein (AKIP1a), one that lacks the third exon (AKIP1b), and one that lacks the third and fifth exon (AKIP1c). AKIP1 is abundantly expressed in cardiac tissue mainly in cardiomyocytes [13]. Therefore, we speculated that AKIP1 was one potential target for miR-320. To test this possibility, we initially conducted a luciferase reporter assay. A fragment of wild-type or mutant 3′ UTR of AKIP1 was cloned into the psiCheck-2 reporter vector (Fig. 3a). Our results showed that miR-320 significantly decreased the firefly luciferase activity of the vector with the wild-type 3′ UTR of AKIP1 cells; however, it had no significant effect on the vector with the mutant 3′ UTR of AKIP1 (Fig. 3b). Furthermore, we detected the mRNA and protein levels of AKIP1 in the miR-320 cardiomyocytes and corresponding control cells, respectively. We determined that both the AKIP1 mRNA and protein levels in the miR-320 cardiomyocytes were significantly lower than the levels in the control group. In contrast, there were significantly higher levels of AKIP1 mRNA and protein in cardiomyocytes transfected with anti-miR-320 mimics compared with the levels in the control group (Fig. 3c and d). Thus, these data demonstrated that miR-320 can directly inhibit AKIP1 expression by binding to its 3′ UTR in cardiomyocytes.

### Expression levels of miR-320 and AKIP1 in myocardial tissue

**Fig. 4:**
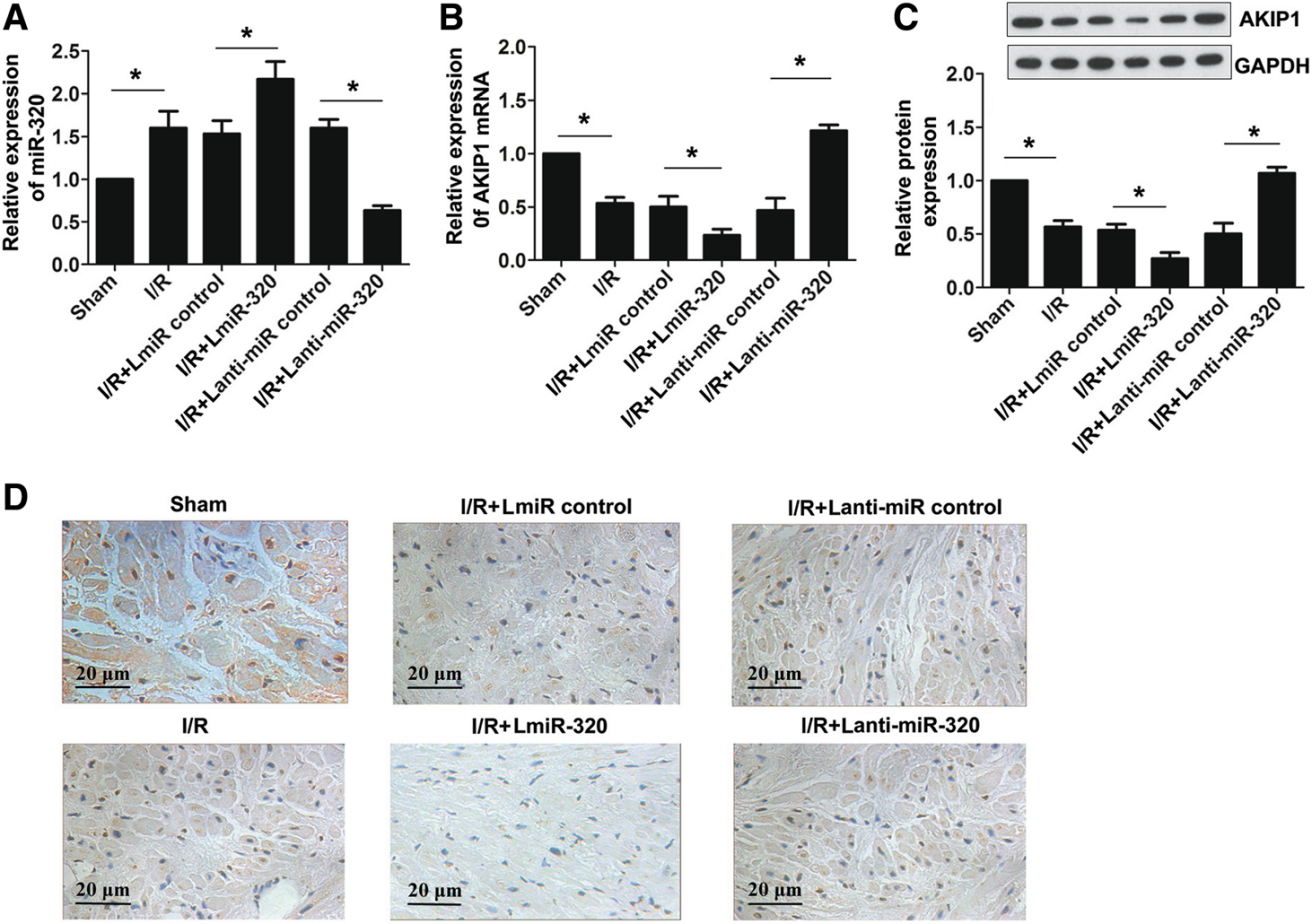
Expression levels of miR-320 and AKIP1 in myocardial tissue. **a**, **b** Relative expression levels of miR-320 and AKIP1 mRNA were evaluated via qRT-PCR in myocardial tissue. **c** Relative protein expression of AKIP1 was detected by Western blot. **d** Immunohistochemistry stain of AKIP1 in myocardial tissue (scale bar = 20 μm). *P < 0.05

In this study, the expression level of miR-320 was remarkably higher in the I/R group compared with the sham group, which suggests increased miR-320 expression after I/R injury in vivo. To assess the correlation between miR-320 and AKIP1 in myocardial tissue, LmiR-320, Lanti-miR-320, and the corresponding negative controls were transfected to myocardial tissue after I/R injury. The miR-320 expression was significantly lower in the Lanti-miR-320 group, whereas it was higher in the LmiR-320 group compared with the corresponding control groups (Fig. 4a). As shown in Fig. 4b and c, compared with the sham group, the AKIP1 mRNA and protein expression was downregulated in the I/R group. The mRNA and protein expression levels of AKIP1 were substantially lower in the LmiR-320 group and significantly higher in the Lanti-miR-320 group compared with the corresponding control groups. A representative immunohistochemistry stain is presented in Fig. 4d. Briefly, these results showed that I/R and H/R treatments may elevate the expression of miR-320 while weakening the expression levels of AKIP1 mRNA and protein, further suggesting that miR-320 inhibits the expression of AKIP1 mRNA and protein.

### MiR-320 performed its role in H9c2 cells subjected to H/R by downregulating AKIP1 expression

**Fig. 5:**
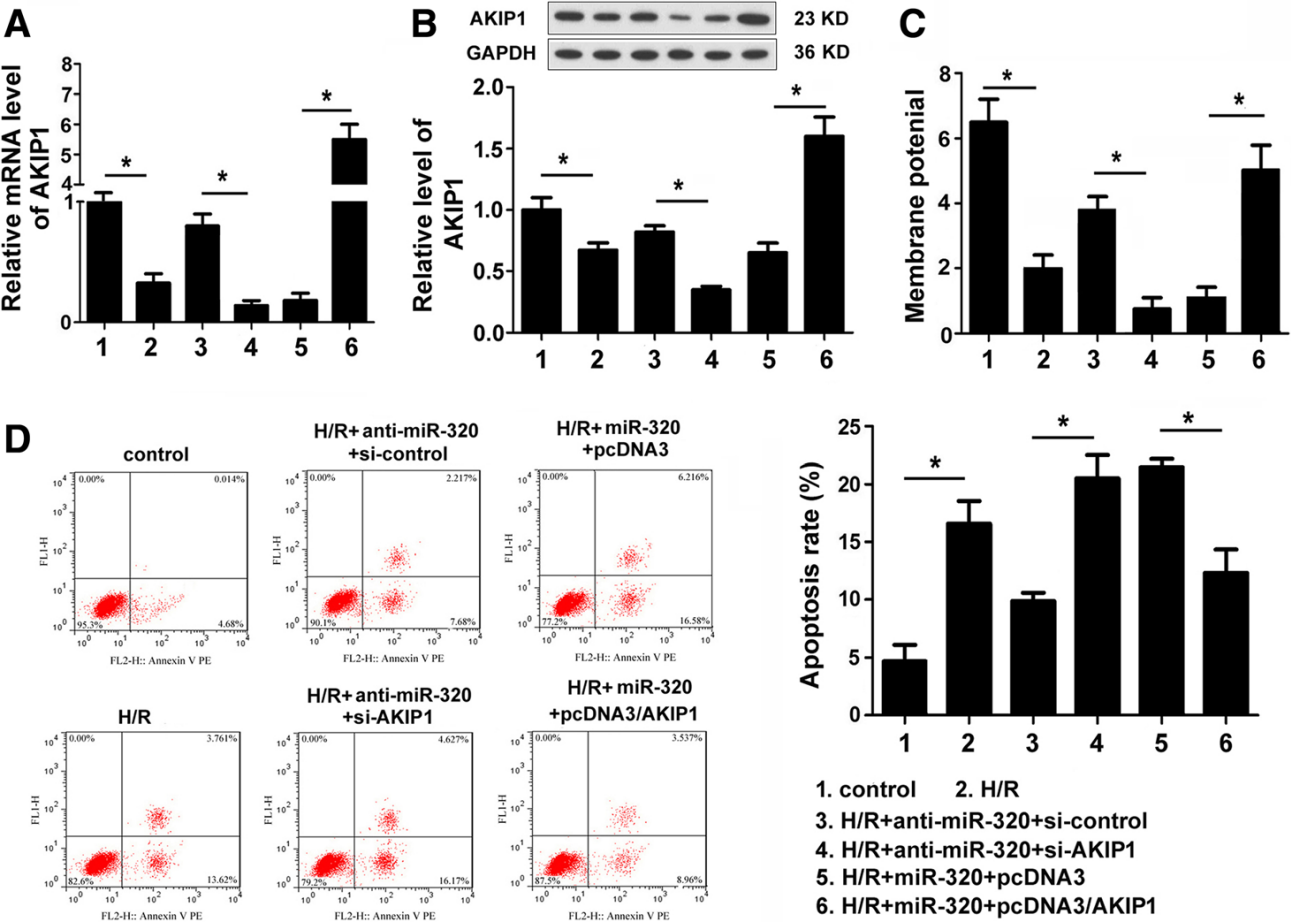
MiR-320 performed its role in H9c2 cells subjected to H/R by downregulating AKIP1 expression. The expression levels of AKIP1 mRNA and protein in H9c2 cells after the co-transfection of anti-miR-320 and si-AKIP1 or miR-320 and pcDNA3/AKIP1 vector were measured via qRT-PCR (**a**) and Western blot (**b**), respectively. **c** The effect of AKIP1 expression on the mitochondrial membrane potential was detected. **d** The effect of AKIP1 on the apoptosis of H9c2 cells that was related to AKIP1 expression was assessed via flow cytometry. *P < 0.05

The AKIP1 protein is involved in cardioprotective effects during in vitro and ex vivo myocardial ischemia [14]. In this research, short hairpin RNA that targeted AKIP1 mRNA (si-AKIP1) was exploited to specifically inhibit the expression of AKIP1 in anti-miR-320 cardiomyocytes after H/R injury. Moreover, the pcDNA3/AKIP1 vector was used to overexpress AKIP1 in miR-320 cardiomyocytes. The protein level of AKIP1 in the cardiomyocytes transfected with both anti-miR-320 mimics and si-AKIP1 was substantially lower than the level in the cardiomyocytes co-transfected with anti-miR-320 mimics and si-control as assessed by Western blot. The relative level of the AKIP1 protein in the cardiomyocytes with both miR-320 mimics and the vector that overexpressed AKIP1 (pcDNA3/AKIP1) was significantly higher than the level in the cells co-transfected with miR-320 mimics and the control vector (Fig. 5a). These results support the idea that miR-320 plays the same role as si-AKIP1 in cardiomyocytes and served as a negative regulator of the AKIP1 expression. As shown in Fig. 5b, the inhibition of AKIP1 expression increased the rate of cardiomyocyte apoptosis, whereas the overexpression of AKIP1 reversed the accelerating effect of miR-320 on cardiomyocyte apoptosis. Moreover, we determined that inhibition of the AKIP1 expression significantly increased the loss of the mitochondrial membrane potential of cardiomyocytes, and the overexpression of AKIP1 significantly attenuated the miR-320-induced enhancing effect on the loss of the mitochondrial membrane potential of cardiomyocytes (Fig. 5c). Taken together, these results suggest that miR-320 promotes cardiomyocyte apoptosis induced by H/R injury partially by targeting AKIP1.

### MiR-320 regulates expression of Bax, Bcl-2, cleaved caspase-3, cleaved caspase-9, p-ASK1, p-JNK and p-p38 in cardiomyocytes after H/R injury by targeting AKIP1

**Fig. 6:**
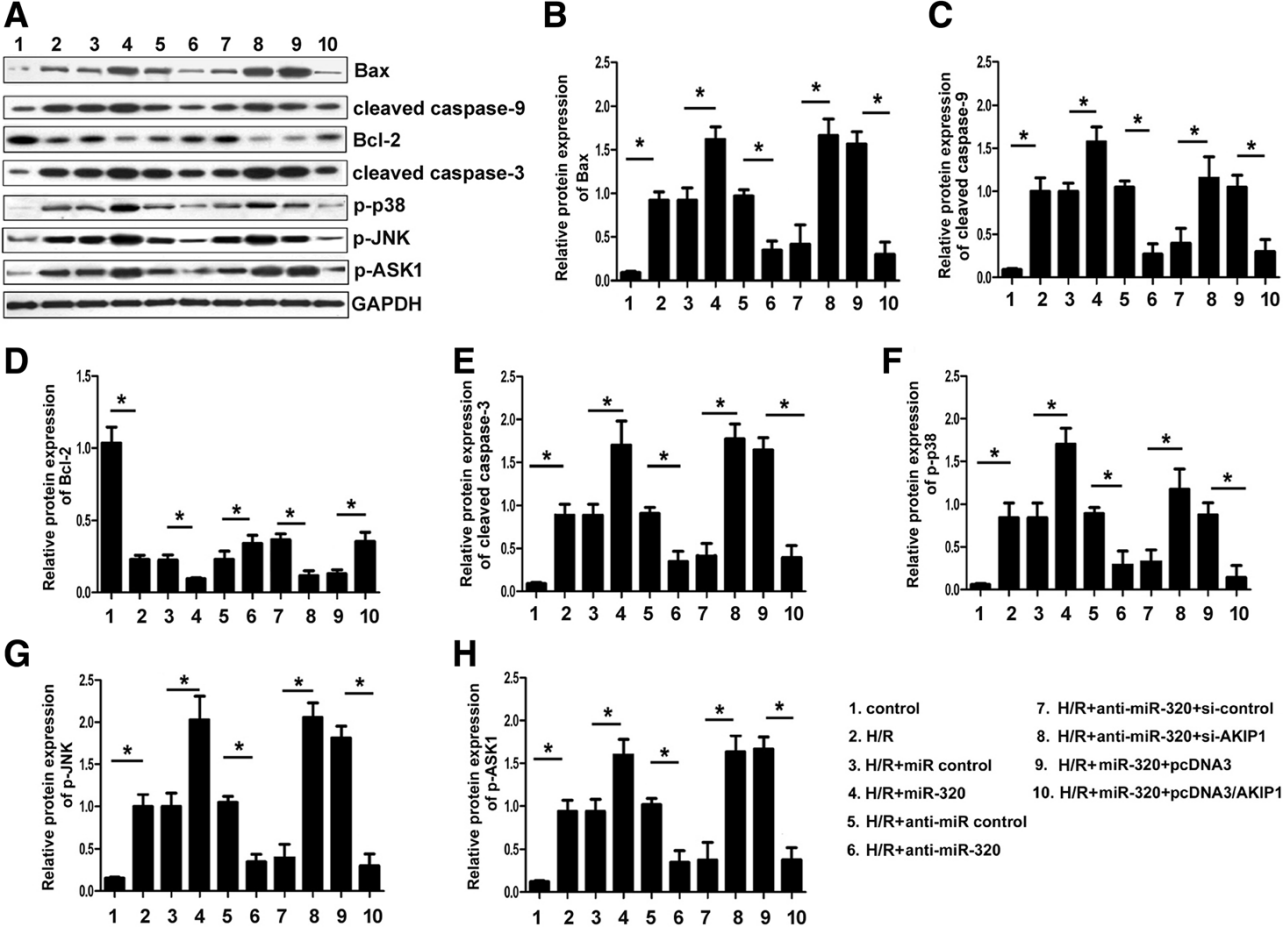
MiR-320 regulated the expression of Bax, Bcl-2, cleaved caspase-3, cleaved caspase-9, p-ASK1, p-JNK and p-p38 in H9c2 cells after H/R injury by targeting AKIP1. H9c2 cells after H/R injury were transfected with miR-320, miR control, miR-320 and pcDNA3/AKIP1 vector, miR-320 and pcDNA3 control vector, anti-miR-320, anti-miR control, anti-miR-320 and si-AKIP1, anti-miR-320 and siRNA control, respectively; the effects of miR-320 on cell apoptosis-related genes (Bax, Bcl-2, cleaved caspase-3, cleaved caspase-9, p-ASK1, p-JNK and p-p38) were analyzed by Western blot. *P < 0.05

Our present results suggest that miR-320 promoted the cardiomyocyte apoptosis induced by H/R injury by targeting AKIP1. To investigate the mechanisms of miR-320 promotion on the apoptosis of cardiomyocytes, we analyzed the effects of miR-320 on the expression of apoptosis-related genes: Bax, Bcl-2, cleaved caspase-3, cleaved caspase-9, p-ASK1, p-JNK and p-p38. The results showed that the downregulation of miR-320 expression by transfecting anti-miR-320 mimics in cardiomyocytes after H/R injury significantly decreased the expression of the pro-apoptosis factors Bax, cleaved caspase-3, cleaved caspase-9, p-ASK1, p-JNK and p-p38, whereas it increased the expression of the anti-apoptosis factor Bcl-2 compared to the controls. However, the overexpression of miR-320 by transfecting miR-320 mimics resulted in the opposite phenomenon. Furthermore, co-transfection with anti-miR-320 mimics and si-AKIP1 vector in cardiomyocytes after H/R injury resulted in increasing expression levels of Bax, cleaved caspase-3, cleaved caspase-9, p-ASK1, p-JNK and p-p38, whereas it decreased the expression of Bcl-2 compared to the controls. In addition, co-transfection with miR-320 mimics and pcDNA3/AKIP1 vector resulted in the opposite phenomenon in cardiomyocytes (Fig. 6).

## Discussion

Recently, microRNAs (miRNAs) have been associated with the regulation of I/R injury-induced cardiomyocyte apoptosis and cardiac function [15]. However, the function and mechanism of these deregulated miRNAs in I/R injury-induced cardiomyocyte apoptosis remain undefined. It has been reported that various microRNAs are deregulated in myocardial I/R injury associated with cell proliferation, apoptosis and death, such as miR-1 [16], miR-21 [17], miR-146a [18], miR-15b [19], miR-195 [20], miR-29 [21], miR-199a-3p and miR-214 [22]. In this study, we initially established a mouse model of myocardial I/R injury and subsequently determined that the miR-320 expression and the rate of cardiomyocyte apoptosis were significantly higher in the I/R injury group and H9c2 cells subjected to H/R compared with the corresponding controls. Moreover, we confirmed AKIP1 as a functional target of miR-320 in H9c2 cells. Thus, our discovery demonstrated that miR-320 may be a promoter of cardiomyocyte apoptosis induced by myocardial I/R injury, and abnormal alteration of the miR-320-AKIP1 interaction may contribute to the occurrence and progression of I/R injury.

Increasing evidence has demonstrated that miR-320 is associated with cell proliferation, apoptosis and death in various pathological processes, including myocardial I/R injury [23–25]. A previous study indicated that miR-320 inhibition using antagomir-320 protects the left ventricle from remodeling after myocardial I/R injury [26]. In this study, we determined that the knock down of miR-320 expression using anti-miR-320 mimics suppressed cell apoptosis and the loss of the mitochondrial membrane potential in H9c2 cells subjected to H/R, whereas the upregulation of miR-320 resulted in the opposite phenomenon as assessed via flow cytometry. On the basis of these results, we considered that miR-320 may serve as a positive regulator in the apoptosis of H9c2 cells subjected to H/R.

MicroRNAs (miRNAs) are a class of small noncoding regulatory RNAs, which broadly regulate target genes by binding to a complementary sequence in their 3′ UTR [27, 28]. Therefore, the establishment of the interrelationship of miRNA and its target genes may provide a better understanding of the molecular mechanism that underlies myocardial I/R injury progression and potential therapeutic targets for the clinical treatment of myocardial I/R injury. Our study showed that the levels of AKIP1 mRNA and protein are both negatively regulated by miR-320 in H9c2 cells. Moreover, the direct targeting was further supported through a luciferase reporter assay. Functional studies have also indicated that the expression level of AKIP1 is negatively correlated with cell apoptosis and the loss of the mitochondrial membrane potential in H9c2 cells subjected to H/R. Thus, these results validate the function of the miR-320-AKIP1 interaction in the progression of myocardial I/R injury.

AKIP1 is a molecular regulator of protein kinase A and nuclear factor kappa B (NF-ĸB) signaling. AKIP1 localizes to the cytoplasm, nucleus, and mitochondria, which suggests that AKIP1 may have multiple functions in the cell, including cell apoptosis [29]. It has recently been reported that AKIP1 stimulates physiological growth in cultured cardiomyocytes and attenuates ischemia/reperfusion (I/R) injury in ex vivo perfused hearts [30]. Moreover, AKIP1 is induced in hypertrophic hearts and may stimulate adaptive cardiomyocyte growth via the Akt pathway [31]. The mitochondrial membrane potential represents an index of the activation of the mitochondrial apoptotic pathway [32]. The mitochondrial stage of apoptosis control is mediated by Bcl-2 activation, and the caspase cascade is thereby activated, thus leading to mitochondrial fragmentation and apoptotic susceptibility [33]. In this study, our results show that the overexpression of AKIP1 may weaken the apoptosis in cardiomyocytes treated with H/R. Furthermore, endomorphin-1 postconditioning may reduce I/R injury and inhibit myocardial cell apoptosis by increasing the Bcl-2/Bax ratio and decreasing the cleaved caspase-3 protein, LDH, IL-6 and CK-MB expression levels in a rat model [34]. The downregulation of ASK1 expression may reduce apoptosis and the myocardial infarct size in a rat model of ischemia/reperfusion [35]. Moreover, it has been reported that cardiomyocyte apoptosis is involved in the myocardial injury of tremor rats by reducing Bcl-2 and p-ERK1/2, upregulating Bax, p-JNK, and p-p38, and activating caspase-3 [36]. In this study, we determined that miR-320 regulates the expression of Bax, Bcl-2, cleaved caspase-3, cleaved caspase-9, p-ASK1, p-JNK and p-p38 in cardiomyocytes after H/R injury by targeting AKIP1. Finally, we observed that both the AKIP1 mRNA and protein levels were negatively correlated with the expression level of miR-320 in myocardial tissue, which suggests that AKIP1 mRNA may be an important miR-320 target in vivo. These results further confirmed that miR-320 played the role of an apoptosis-promoting factor through the negative regulation of AKIP1 in the progression of myocardial I/R injury.

## Conclusions

Our results indicated that miR-320 was significantly upregulated in cardiac muscle tissues after I/R injury and demonstrated the effects of miR-320 on promoting the apoptosis of H9c2 cells subjected to H/R, at least in part by negatively regulating AKIP1 and inducing the mitochondrial apoptotic pathway. These findings suggest that the inhibition of miR-320 protects against myocardial I/R injury and thus provide a possibility to treat I/R injury using a novel strategy in the future.

## Additional file

Additional file 1: Results of miRNA microarray. (XLSX 21 kb)

## Abbreviations

AKIP1: A kinase interacting protein 1
H/R: hypoxia/reoxygenation
I/R: ischemia-reperfusion
IHD: ischemic heart disease
LAD: left anterior descending
PBS: phosphate buffer solution
qRT-PCR: quantitative real-time polymerase chain reaction
TUNEL: terminal dUTP nick end-labeling
